# First‐in‐human high‐cumulative‐dose stem cell therapy in idiopathic pulmonary fibrosis with rapid lung function decline

**DOI:** 10.1002/sctm.19-0037

**Published:** 2019-10-15

**Authors:** Alexander Averyanov, Irina Koroleva, Mikhail Konoplyannikov, Veronika Revkova, Victor Lesnyak, Vladimir Kalsin, Olesya Danilevskaya, Alexey Nikitin, Anna Sotnikova, Svetlana Kotova, Vladimir Baklaushev

**Affiliations:** ^1^ Federal Research and Clinical Center of Federal Medical‐Biologic Agency Moscow Russia; ^2^ Pulmonology Scientific Research Institute under Federal Medical‐Biologic Agency Moscow Russia; ^3^ Institute for Regenerative Medicine I. M.Sechenov First Moscow State Medical University Moscow Russia; ^4^ Semenov Institute of Chemical Physics Moscow

**Keywords:** adult human bone marrow, adult stem cells, bone marrow stromal cells, cellular therapy, clinical trials, lung, stem cell transplantation, transplantation tolerance

## Abstract

Previous phase I studies demonstrated safety and some beneficial effects of mesenchymal stem cells (MSCs) in patients with mild to moderate idiopathic pulmonary fibrosis (IPF). The aim of our study was to evaluate the safety, tolerability, and efficacy of a high cumulative dose of bone marrow MSCs in patients with rapid progressive course of severe to moderate IPF. Twenty patients with forced ventilation capacity (FVC) ≥40% and diffusing capacity of the lung for carbon monoxide (DLCO) ≥20% with a decline of both >10% over the previous 12 months were randomized into two groups: one group received two intravenous doses of allogeneic MSCs (2 × 10^8^ cells) every 3 months, and the second group received a placebo. A total amount of 1.6 × 10^9^ MSCs had been administered to each patient after the study completion. There were no significant adverse effects after administration of MSCs in any patients. In the group of MSC therapy, we observed significantly better improvement for the 6‐minute walk distance in 13 weeks, for DLCO in 26 weeks, and for FVC in 39 weeks compared with placebo. FVC for 12 months in the MSCs therapy group increased by 7.8% from baseline, whereas it declined by 5.9% in the placebo group. We did not find differences between the groups in mortality (two patients died in each group) or any changes in the high‐resolution computed tomography fibrosis score. In patients with IPF and a rapid pulmonary function decline, therapy with high doses of allogeneic MSCs is a safe and promising method to reduce disease progression.

## Graphical abstract

High cumulative dose of mesenchymal stem cells is safe and well tolerated by patients with severe idiopathic pulmonary fibrosis. The patients in the main group during 12 months follow‐up demonstrated absence of significant adverse effects, and had their lung function significantly better than in placebo group.

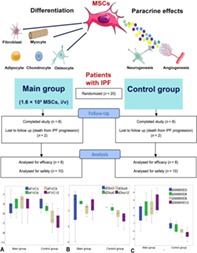

**Lessons learned**

- The primary objective was the evaluation of the safety and tolerability of repeated infusions of high doses of bone marrow‐derived MSCs up to the total cumulative dose of 2 billion cells in subjects with rapidly progressing idiopathic pulmonary fibrosis.
- The evaluation was based on the number and severity of AEs related to the infusion during 52 weeks of follow‐up.
- The secondary objective was evaluation of the main lung function parameters, such as forced ventilation capacity and diffusing capacity of the lung for carbon monoxide.
- The stem cell treatment has been found safe and well tolerable.
- Patients in the main group had their lung function increased, as compared to the placebo group, in which the continued decline of the lung function was observed.
- These findings allow us to conclude that such stem cell therapy is effective for the treatment of rapidly progressing idiopathic pulmonary fibrosis.

**Significance statement**

The results of this first‐in‐human clinical trial revealed that a high cumulative dose of mesenchymal stem cells (MSCs) is safe and well tolerated by patients with idiopathic pulmonary fibrosis with a rapid lung function decline. During the treatment period, the patients in the main group experienced increased lung function; however, the patients in the placebo group experienced a continued decline in lung function. Thus, this study shows the safety, tolerability, and potential benefits of greater doses of MSCs than those used earlier in patients with idiopathic pulmonary fibrosis, and these findings might move future trials toward a new step in stem cells transplantation.

## 1 INTRODUCTION

Idiopathic pulmonary fibrosis (IPF) is the most common of the interstitial lung diseases, a progressing chronic disease with unknown origin characterized by the development of fibrotic transformation of the lung parenchyma, predominantly in the older population.^1^ The prevalence of IPF appears to be increasing, potentially explained by population aging.^2^ The survival median of patients with IPF is 3 to 5 years; at the same time, the individual survival of patients may be extremely variable.^3^, ^4^, ^5^ Conventionally, the IPF clinical course can be categorized into three types based on the rate of the forced ventilation capacity (FVC) decline: slow deterioration (less than 10% decline in FVC over 6 months or no changes in the disease indicators for many months), intermittent deterioration with episodes of exacerbations and rapid deterioration (more than 10% decline in FVC for 6 months).^3^, ^6^, ^7^ For the last type, before the era of antifibrotic medications, the life expectancy of patients did not usually exceed 2 years from the time of diagnosis.^6^, ^7^, ^8^ Besides acute exacerbations, a poor prognosis might be suspected in patients with IPF with such predictors as low values of FVC and diffusing capacity of the lung for carbon monoxide (DLCO), old age, and a high rate of pulmonary function decline.^3^, ^9^ The majority of clinical studies on the drug therapy of IPF did not evaluate the pretreatment functional reduction rate^10^, ^11^; therefore, it is difficult to judge its efficiency in patients with a fast lung function decline.

After a large number of successful preclinical studies, a treatment with mesenchymal stem cells (MSCs) is considered a potential new direction for lung fibrosis therapy.^12^, ^13^ The potential effects of MSCs in pulmonary fibrosis are related to their ability to produce a large number of biologically active substances with anti‐inflammatory, immunosuppressive, and angiogenic properties.^12^ The results of the published clinical trials on the cell therapy for IPF confirmed the safety of MSCs; however, all of these studies included patients with mild to moderate impairment of lung function, without taking into account the rate of their preceding decline. Also, the total dose of administered cells did not exceed 2 × 10^8^.^14^, ^15^, ^16^ We performed the first clinical trial in this field in patients who had a fast lung function decline, using a significantly more intensive intervention with respect to both the individual MSCs dose and the frequency of dose administration.

## 2 MATERIALS AND METHODS

### 2.1 Study design

The phase I/IIA study reported here was randomized, open‐label, and placebo‐controlled in two groups and was conducted at the Federal Research Clinical Center of Federal Medical‐Biologic Agency of Russia. The study was approved by the local ethical committee (approval 5‐11‐03‐2013 of March 03, 2013) and registered at http://clinicaltrials.gov (identifier NCT02594839).

Twenty patients were included into the study (age 33‐74 years, 11 males, 9 females), all of whom had a histologically or radiologically confirmed pattern of usual interstitial pneumonia (UIP), with a history of a lung function decline (FVC or DLCO) ≥10% over the last 12 months and a current FVC ≥40% of predicted and DLCO ≥20% of predicted. The complete list of inclusion/exclusion criteria is shown in Supporting Information Appendix S1. According to the study protocol, those patients that met the eligibility criteria were randomized into two groups, with 10 patients per group: group 1 received four series of MSCs intravenous infusions, repeated after 12 weeks. Each of the series included two intravenous infusions performed in a 7‐day interval. Each infusion contained a suspension of 200 million cells in 400 mL of normal saline. The last MSCs administration was performed at 39 weeks after the study beginning. The total number of MSCs administered in group 1 was 1.6 × 10^9^ for the patients who completed the protocol. Group 2 received a placebo (400 mL of normal saline) with the same schedule. Sixteen patients completed the study (Figure 2).

The total characteristics of enrolled patients in each group are presented in Table 1. None of the patients took antifibrotic drugs because of their inaccessibility in the country during the study. Most of patients took prednisone as a single drug approved by the current national guidelines. The diagnosis of IPF was based on the results of a multidisciplinary discussion (MDD) according to American Thoracic Society/European Respiratory Society criteria.^1^ The primary endpoint was the evaluation of treatment‐related adverse events (AEs), related to timing of each dosing regimen of the MSCs suspension. Vital signs (including body temperature, pulse rate, respiration rate, and blood pressure), oxygen saturation, 12‐lead electrocardiogram, and clinical laboratory tests (including hematology, chemistry, and urinalysis) were assessed before and during 3 days after each infusion. The AE frequency and severity were coded according to the *Medical Dictionary for Regulatory Activities*, version 16.1. All AEs were recorded from the time the subject signed the informed consent until 12 weeks after the last dose of MSCs or placebo. The secondary endpoints were as follows: (a) changes in the lung function (FVC, DLCO), (b) physical tolerance (6‐min walk test distance (6MWTD) at week 13, 26, 39, and 52 of the therapy, and (c) a semiquantitative estimation of pulmonary fibrosis by high‐resolution computed tomography (HRCT) data at week 26 and 52. Spirometry and diffusion tests were conducted in accordance with european respiratory society (ERS)/american thoracic society (ATS) regulations.^17^, ^18^ The 6MWTD was performed according to the АТS protocol.^19^ A semiquantitative assessment of the HRCT pulmonary fibrosis score was performed by two independent radiologists using the method of Oda et al.^20^

**Table 1:** Descriptive statistics for the groups at the baseline point*

Group	Sex	Age (years)	Disease duration (months)	BMI (kg/m^2^)	Prednisone (dose mg/daily)	FVC (% of predicted)	DLCO (% of predicted)	UIP biopsy proved	Smoking history
*Group 1: MSC treatment*									
1	M	61	21	27	20	67	41	+	+
2	F	60	30	23	20	75	22	+	+
3	M	50	27	30	25	36	24	+	−
4	F	74	51	19	0	79	28	−	−
5	F	58	84	18	10	43	28	−	+
6	M	33	16	27	15	52	31	+	+
7	M	68	27	25	10	48	33	+	−
8	M	61	45	23	20	55	37	−	+
9	F	59	48	22	20	51	22	+	−
10	F	70	66	19	25	40	20	+	+
Summary	5/5	59.4 ± 11.5*	41.5 ± 21.5*	23.3 ± 4.0*	16.5 ± 7.8*	54.6 ± 14.6	28.6 ± 6.9	7*	6*
*Group 2: Placebo*									
1	M	56	30	20	20	61	38	+	+
2	F	59	18	26	25	53	29	−	−
3	F	65	21	26	20	48	26	+	−
4	M	69	51	24	15	61	35	+	−
5	M	52	24	28	12.5	50	31	+	−
6	M	71	45	20	15	45	24	−	+
7	M	69	48	26	15	52	30	−	+
8	F	60	54	29	15	64	39	+	−
9	M	58	18	21	25	45	21	+	+
10	F	66	12	17	25	49	21	−	−
Summary	6/4	62.5 ± 6.4*	32.1 ± 15.8*	23.7 ± 4.0*	18.8 ± 4.9*	52.8 ± 6.9*	29.4 ± 6.5*	6*	4*

*Note*: Data are presented as mean **±** SD.

Abbreviations: BMI, body mass index; DLCO, diffusing capacity of the lung for carbon monoxide; F, female; FVC, forced ventilation capacity; M, male; UIP, usual interstitial pneumonia.

* There were no significant differences between the study groups (*P* > .05, using general linear model).

### 2.2 MSCs preparation

#### 2.2.1 Isolation of human MSCs

For half of the patients (n = 5), the bone marrow for MSCs separation was obtained from young (20‐35 years of age), healthy blood relatives. In cases of the absence of young, healthy relatives or their refusal (n = 5), we used the bone marrow from healthy donors with the same sex as the patient. In these cases, donors for each MSC injection were randomized. Each donor signed the informed consent, and the protocol of bone marrow harvesting was approved by the local ethics committee of the Federal Research Clinical Center of FMBA of Russia (4‐11‐03‐2013 of March 03, 2013).

A total of 100 to 150 mL of bone marrow was collected from donors pretested for cytomegalovirus (CMV), human immunodeficiency virus 1 (HIV‐1) and 2, human T‐lymphotropic virus 1 (HTLV‐1) and 2, Epstein–Barr virus (EBV), B19, hepatitis B virus, and hepatitis C virus into heparin‐containing test tubes (100 U/mL of punctate; Sigma, Sigma‐Aldrich Corp., St. Louis, Missouri). MSCs were obtained from bone marrow‐derived mononuclear cells according to the standard protocol.^21^

#### 2.2.2 Flow cytometry analysis

MSCs (passage 3‐5) were washed with phosphate‐buffered saline containing 1% fetal bovine serum (FBS). Fluorescein isothiocyanate‐conjugated anti‐human CD34, CD45, and CD105 antibodies and phycoerythrin‐conjugated antihuman CD29, CD 44, CD73, and CD90 antibodies were used for staining the cells. All antibodies were purchased from Miltenyi Biotec. The analysis was performed with a CyFlow Space flow cytometer (Sysmex Partec) using the Partec FloMax flow cytometry Data Acquisition and Analysis Software.

#### 2.2.3 Osteogenic, adipogenic, and chondrogenic differentiation

The cultured MSCs (passage 5) were differentiated into the osteogenic, adipogenic, and chondrogenic lineage by culturing in the osteogenic medium (Dulbecco's modified Eagle's medium [DMEM] supplemented with 10^−8^ M dexamethasone [Sigma, D4902], 10 mM b‐glycerophosphate [Sigma, G9422], and 50 μg/mL ascorbic acid), adipogenic medium (DMEM supplemented with 10 mM 3‐isobutyl‐1‐methylxanthine [Sigma, 17018], 0.1 mM indomethacin [Sigma, 17378], 10 μg/mL insulin [Sigma, I6634], 10^−6^ dexamethasone), and chondrogenic medium (Stempro, Invitrogen, Life Technologies Ltd Paisley PA4 9RF, UK), with the subsequent staining with alizarin red (Sigma), Oil Red O (Sigma), and Alcian blue (Sigma), respectively. A Nikon Eclipse Ci microscope (Nikon Instruments Inc., New York) was used for the image capture.

#### 2.2.4 Karyotyping

Karyotypes were analyzed in MSCs from the first and the last (4 or 5) passages that were isolated and cultured in vitro using the chromosome multicolor fluorescence in situ hybridization (mFISH) technique.^22^ The cells were incubated for 4 h with colchicine (40 μg/mL), and then the cells were incubated in 0.075% KCl fixed with Carnoy's solution (3:1 vol/vol absolute ethanol: glacial acetic acid), dropped onto slides, and dried. mFISH for chromosome karyotyping visualization was then performed using the 24 XCyte color kit (Meta‐Systems, Altlussheim, Germany) according to the manufacturer's recommendations.

#### 2.2.5 MSC preparation for their injection to patients: Management of cell therapy‐related risks

At the first stage of the study, we performed an exam of each donor for the absence of bloodborne diseases (HIV‐1 and ‐2; hepatitis C virus; hepatitis B, D, and E; syphilis; CMV, EBV, HTLV, acute myeloid leukemia, and chronic myeloid leukemia) to comply with the safety requirements.

For injection to patients, MSCs of a third to fifth passage were used, characterized by CD markers, karyotype, sterility tests, and apyrogenicity (for each MSC series). The preparations' sterility was tested via the seeding on culture media and using the matrix‐assisted laser desorption/ionization (MALDI)‐time‐of‐flight mass spectroscopy analysis (Microflex LRF, Bruker) in the federal research and clinical center (the main affilation) bacteriological laboratory. For the mass spectroscopy, we used MALDI Sepsityper Kit, modified for the conditioned media (CM). Briefly, all CM from MSCs was collected and incubated for 5 days in bacteriological thermostat. At the same time, the samples of CM were seeded in the bacteriological media. Additionally, the cells from each batch were propagated up to 10^9^, centrifuged, and prepared for mass spectroscopy according to the manufacturer protocol. The taxonomic dataset consist of 7854 spectra of prokaryotic and fungi organisms. No cases of microbial or fungal contamination were detected. Although the contamination has not been detected by mass spectroscopy, the CM samples from each cell batch were also tested using chromogenic limulus amebocyte lysate‐test (Hycult biotech, HIT302), which is more sensitive for bacterial endotoxin detection. In each case, the level of endotoxin was less than 0.1 UE/mL.

In order to minimize the negative effect of the medium components, MSCs were cultured without the FBS addition; instead, the autologous serum and platelet lysate were used. The platelet lysate was prepared from the patients' plasma by freezing–thawing of the platelet fraction. The cells were kept in the hypoxic (5% О_2_) conditions for the entire duration of culturing that, besides the activation of glycolytic processes characteristic of multipotent stem cells, allowed minimization of oxidative damage to the cell membrane systems.

When making each preparation, the following was controlled: (a) cell concentration (2 × 10^6^ for each preparation); (b) survival—using a Luna 2 cell counter (it was no less than 95% in each preparation directly before the injection); and (c) the absence of mycoplasma contamination (using the real‐time PCR analysis, Mycoplasma Detection Kit; ThermoFisher).

### 2.3 Statistical analysis

A statistical power analysis showed 76% and 94% for FVC and DLCO, respectively, based on a sample size of 20 patients in two comparable groups. The primary efficacy endpoint of the study was the rate of decline in percent of predicted FVC value from baseline to week 52. A generalized linear repeated measures model was used as the primary efficacy analysis. The secondary efficacy endpoints of the study were changes in percent of predicted DLCO value, 6MWD test (meters), and chest HRCT fibrosis score (points) from baseline to week 52. Another secondary efficacy endpoint included the change in a rate of FVC decline (percentage of the predicted value) from 12 months before the treatment to baseline and from baseline to week 52. To compare the study groups, we used multivariate tests and nonparametric Mann‐Whitney *U* test. The interobserver agreement for the HRCT‐based fibrosis score was calculated in accordance with the recommendations by Kundel and Polansky, using Cohen's kappa coefficient with 95% confidence limits.^23^ A safety analysis was performed in the safety population. All AEs were compared in two groups by Fisher's exact criteria. A two‐sided probability threshold of .05 was considered statistically significant. The descriptive data are presented as mean ± SD and the other data as median and interquartile range.

## 3 RESULTS

### 3.1 MSCs characterization

**Figure 1:**
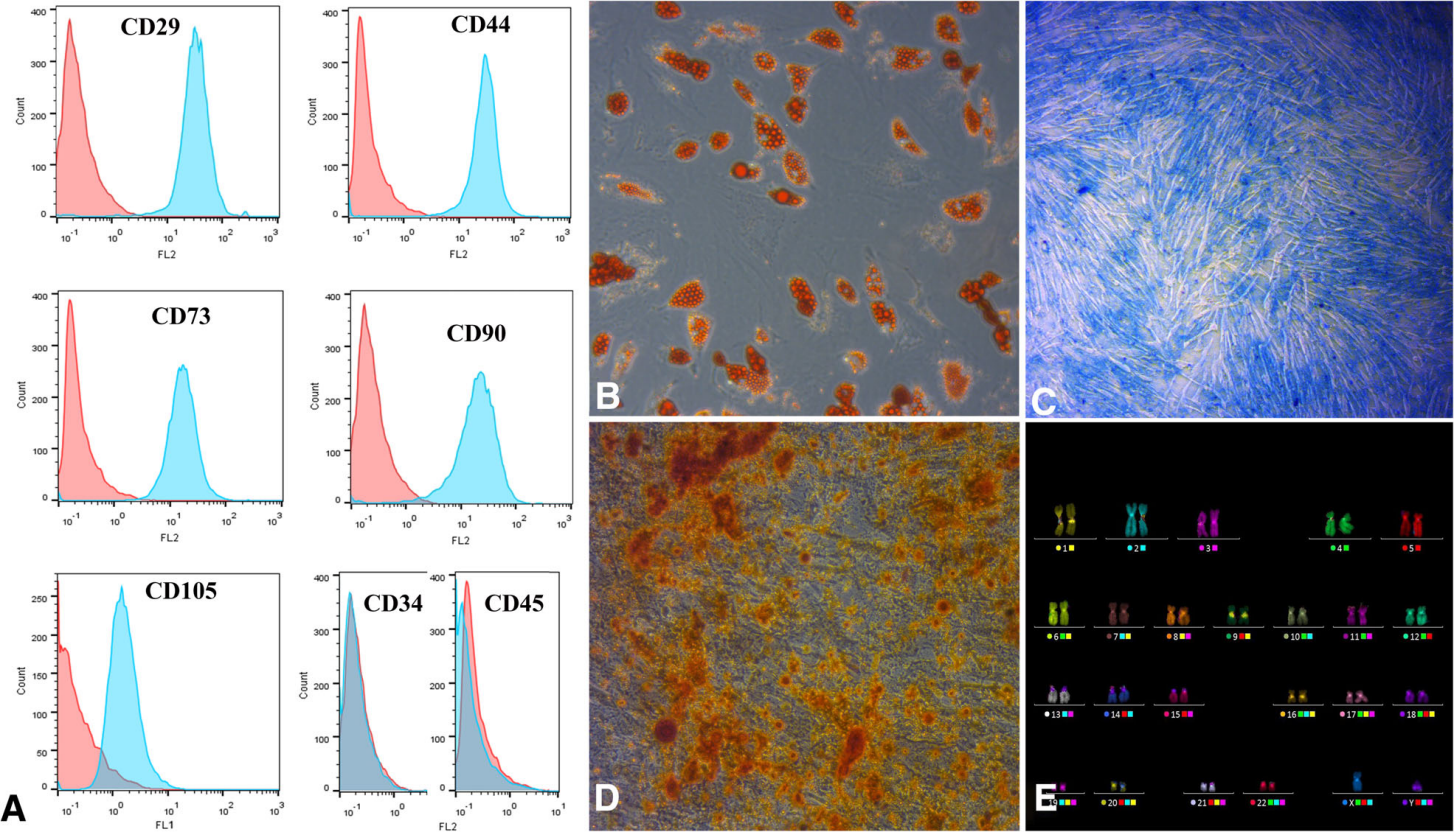
Characterization of cultured human bone marrow‐derived mesenchymal stem cell (MSCs; passage 4) before the transplantation. A, CD‐immunophenotyping of MSCs using flow cytometry. Red histograms represent isotype specific Ig control; blue histograms: fluorescein isothiocyanate/PE‐conjugated antihuman CD antibodies. B‐D, Differentiation analysis. MSCs were characterized by their differentiation potential by staining with Oil Red O—adipogenic lineage, B, Alcian blue—specific to sulfated GAGs, C, and alizarin red—osteogenic lineage, D. Magnification ×200, B‐D. E, Karyotype analysis of human MSCs, mFISH visualization method

The results from the flow cytometric analysis revealed that the standardized culture of human MSCs in vitro resulted in stable expression of the surface markers following a serial passage. The flow cytometry analysis showed that the cultured MSCs had a CD29^+^ CD44^+^ CD73^+^ CD90^+^ CD105^+^ CD34^−^ CD45^−^ phenotype, the one characteristic of adult human MSCs (Figure 1A). The differentiation potential of MSCs was identified by their osteogenic, adipogenic, and chondrogenic differentiation and demonstrated by positive staining with alizarin red, Oil Red O, and Alcian blue, respectively (Figure 1B‐D). A normal diploid karyotype with 46 chromosomes and no abnormal changes in the chromosome structure was observed by the analysis of 30 metaphase cells by mFISH visualization method (Figure 1E).

**Figure 2:**
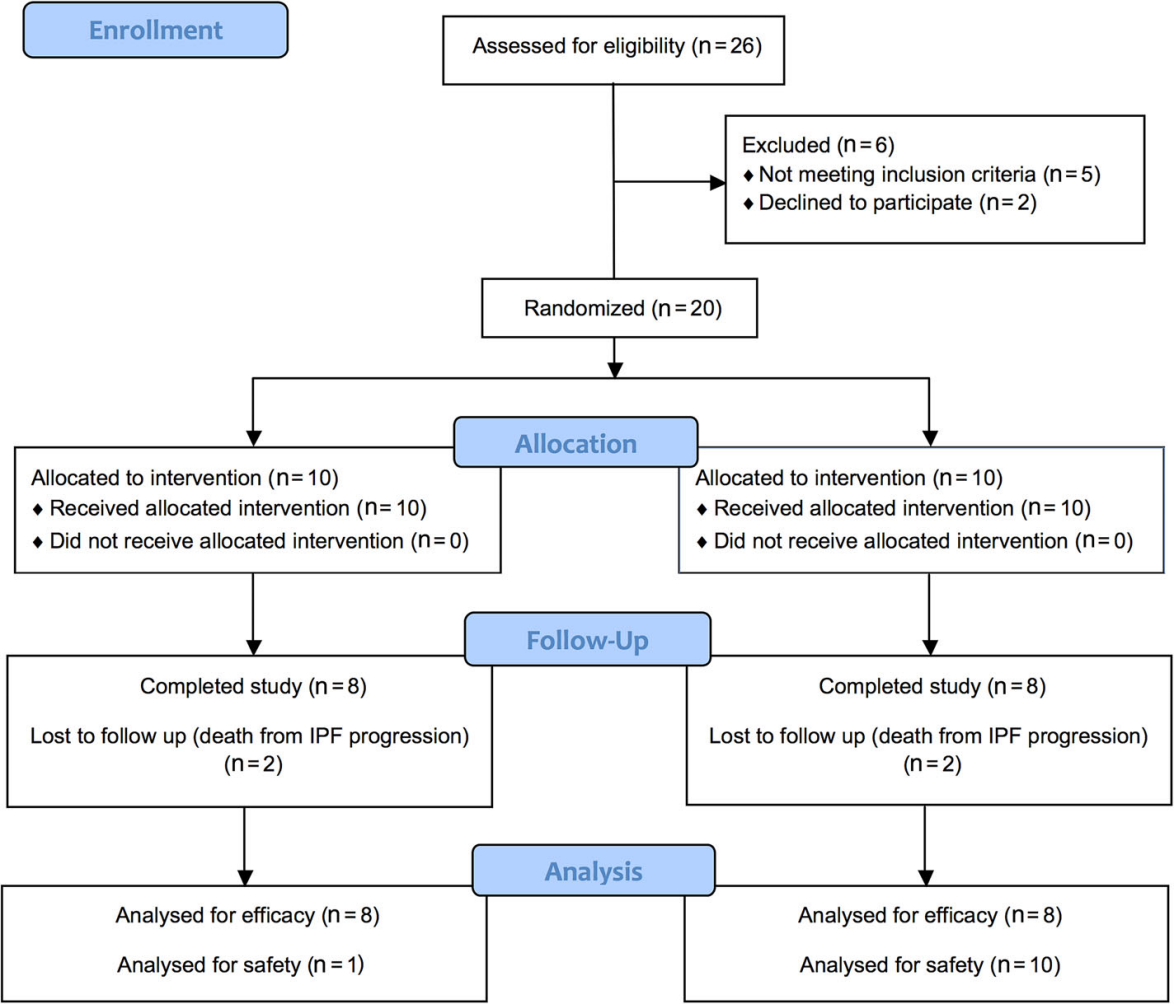
CONSORT flow diagram

**Figure 3:**
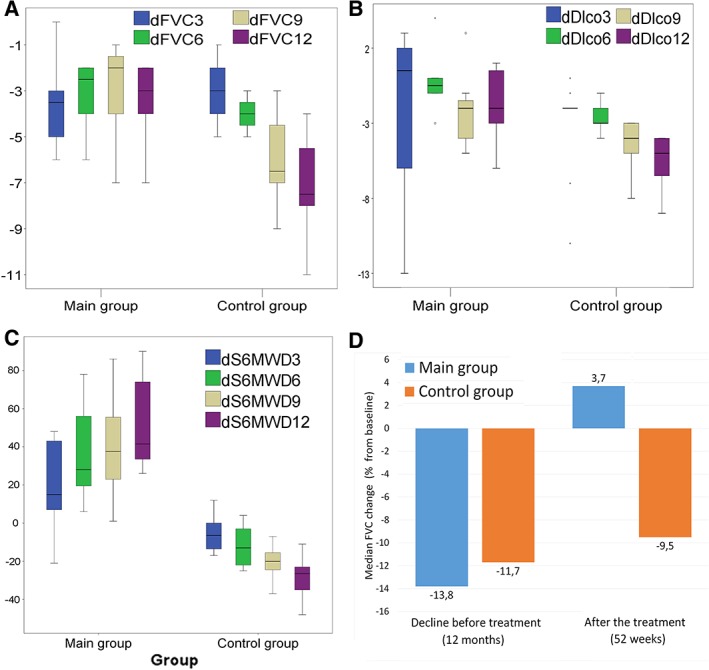
Secondary endpoints dynamics. A‐C, Differences (delta) of real values (*y*‐axis) of forced ventilation capacity (FVC; A), diffusing capacity of the lung for carbon monoxide, B, and 6MWD, C, from the baseline level, during the treatment period (median, minimum and maximum, 25% and 75% quartiles are shown). D, Change from the baseline in FVC median (% of predicted) during 12 months before the treatment and during the treatment (by week 52) in the main group and the control. In the legend, 3, 6, 9, and 12 mean the values after 3, 6, 9, and 12 months, respectively

Thus, the characterization of cells prior to administration revealed that we obtained three potent MSCs with normal diploid karyotype and a high level of expression of MSC‐specific surface markers.

### 3.2 Safety

Of the 20 initially recruited patients, 16 completed the study. Four patients (patients 9 and 10 from each group, 3 females and 1 male) died from respiratory failure progression due to IPF (one was autopsied) between weeks 14 and 34. All of these four patients had initially low indices of DLCO (20%‐22% of predicted), FVC (40%‐51%), and 6MWTD (89‐150 m) and high scores of lung fibrosis according to the HRCT data (167‐190 points), and they took higher doses of prednisone (20‐25 mg daily) than other patients did.

Adverse effects were more frequently observed in the group receiving MSCs; among them were fever and chills, predominantly in the first day after infusion (Table 2). However, these reactions were mild and did not require study discontinuation. Light fever after an intravenous cell infusion was noted at least once over the whole study duration in 4 out of 10 patients. One female patient in the MSCs therapy group developed an ischemic stroke, with almost all sensory and motor functions restored after anticoagulant and vascular therapy. In the placebo group 1, one male patient suffered an exacerbation of IPF; no exacerbations were detected in the MSCs group. The statistical analysis did not reveal any therapy‐associated significant AEs (Table 2).

**Table 2:** Adverse effects

Events	Subjects, n (%)/events, n			*P* value
	1	2	Total	
	(n = 10)	(n = 10)	(n = 20)	
All AEs	8 (80.0) / 28	7 (70.0) / 16	15 (75.0) / 44	>.999^
Death	2 (20.0) / 2	2 (20.0) / 2	4 (20.0) / 4	>.999^
Transient fever	4 (40.0) / 6	1 (10.0) / 1	5 (25.0) / 7	.303^
Weakness	4 (40.0) / 4	1 (10.0) / 1	5 (25.0) / 5	.303^
Cough	2 (20.0) / 2	2 (20.0) / 2	4 (20.0) / 4	>.999^
Headache	2 (20.0) / 4	2 (20.0) / 2	4 (20.0) / 6	>.999^
LRTI	2 (20.0) / 2	2 (20.0) / 2	4 (20.0) / 4	>.999^
Nausea	2 (20.0) / 2	2 (20.0) / 2	4 (20.0) / 4	>.999^
URTI	2 (20.0) / 3	2 (20.0) / 2	4 (20.0) / 5	>.999^
Chill	2 (20.0) / 2	0 (0.0) / 0	2 (10.0) / 2	.474^
IPF exacerbation	0 (0.0) / 0	1 (10.0) / 1	1 (5.0) / 1	>.999^
Ischemic stroke	1 (10.0) / 1	0 (0.0) / 0	1 (5.0) / 1	>.999^
Skin rash	0 (0.0) / 0	1 (10.0) / 1	1 (5.0) / 1	>.999^

*Note*: There were no significant differences between the study groups compared using the general linear model.

Abbreviations: AEs, adverse events; IPF, idiopathic pulmonary fibrosis; LRTI, lower respiratory tract infection; URTI, upper respiratory tract infection.

### 3.3 Overall effects of MSCs versus placebo

After the first 3 months of treatment (administration of 400 million MSCs), we did not observe a statistically significant difference between the groups with regard to any of the studied indices, except for the 6MWTD, which increased by 15.3% in the MSCs group and decreased by 2.7% in the placebo group (Table 3). By the sixth month of the therapy, the MSCs group showed a significantly better DLCO change (−1.2% in the MSCs group vs −6.5% in the placebo group) and 6MWTD (+22.0% vs −3.4% in the placebo group) compared with baseline. After 9 months, and a total cell dose of 1.2 × 10^9^, the difference in the dynamics from baseline points was observed in FVC (+4.7% vs −7.6%) and 6MWDT (+24.2% vs −6.7%). After the treatment completion and reaching the total dose of 1.6 billion MSCs after 12 months (by week 52), statistically significant differences were observed in the dynamics of FVC (+3.7% vs −9.5%), DLCO (−5.1% vs −12.9%), and 6MWDT (+26.6% vs −9.8%) from the baseline (Figure 3A‐C).

**Table 3:** Dynamics of basic study points

MSCs, Placebo	12 months before	Baseline	13th week	26th week	39th week	52nd week
*FVC (% of predicted)*						
MSCs	60.0 (52‐72)	51.5 (43‐67)	46.5 (41‐62)	51.5 (44‐67)	56* (43‐69)	55.5* (42‐68)
Placebo	58.0 (55‐68)	51.0 (48‐61)	49.0 (45‐56)	49.5 (45‐57)	49.0 (44‐53)	48.0 (43‐52)
*DLCO (% of predicted)*						
MSCs	29.0 (26‐42)	28.0 (22‐33)	28.5 (24‐34)	29.0* (25‐36)	28.0 (22‐34)	28.0* (24‐34)
Placebo	30.0 (27‐40)	29.5 (24‐35)	27.5 (22‐34)	28.5 (24‐34)	27.5 (23‐31)	26.5 (22‐29)
*6MWDT distance (m)*						
MSCs	ND	271.0 (150‐381)	340* (240‐390)	360** (261‐423)	366.5** (270‐404)	373.5** (273‐429)
Placebo	ND	247.5 (197‐346)	289 (229‐377)	287 (223‐370)	277 (220‐362)	267.5 (212‐359)
*HRCT score (points)*						
MSCs	ND	150.5 (135‐172)	ND	143.5 (134‐157)	ND	145.0 (135‐165)
Placebo	ND	138.5 (130‐149)	ND	136.5 (131‐146)	ND	138.0 (133‐143)

*Note*: Data are presented as median (interquartile range).

Abbreviations: 6MWTD, 6‐minute walk test difference; DLCO, diffusing capacity of the lung for carbon monoxide; FVC, forced ventilation capacity; HRCT, high‐resolution computed tomography; ND, no data.

* Statistically significant differences (*P* < .05) between the study groups.

** Statistically significant differences (*P* < .01) between the study groups.

The interobserver agreement (*κ* value) in the HRCT signs estimation was in general high and determined as 0.69 for the reticular changes, 0.75 for the traction bronchiectasis, 0.85 for the normal attenuation, and 0.88 for the honeycombing. The HRCT fibrosis score did not differ significantly from the baseline by week 26 (0.7% for MSCs vs 0% for placebo) and by week 52 (1.7% for MSCs vs 0.7% for placebo).

When evaluating the FVC decline for the 12 months preceding randomization and during the 52 weeks of the therapy, we found a significant improvement in the group receiving MSCs (from −13.8% to +3.7%) as compared with the placebo group (from −11.7% to −9.5%; Figure 3D). We analyzed individual patients in the MSCs therapy group, who had a continuing decline of FVC and DLCO >10% over the observation period (patients 4 and 5), as well as deceased patients (patients 9 and 10). All patients with no response to the therapy were females, had the longest disease history (48‐84 months), and had low body mass index (BMI; three out of four had a BMI of 18 to 19 kg/m^2^, one a BMI of 22 kg/m^2^) and a low initial level of DLCO (<30% of predicted).

## 4 DISCUSSION

The presented clinical study was the first one in which the effects of intravenous MSCs transplantation in patients with rapidly progressing IPF were compared with the effects of a placebo and significantly higher and more frequent doses of cell infusions than those in the previous investigations were applied. The study demonstrated the safety of a total dose of 1.6 × 10^9^ cells, which all the participants of active group having completed the study received. Although the incidence of side effects was more frequent in the MSC therapy group than in the placebo group, they did not have severe manifestations, and the statistical analysis using Fisher's exact criteria did not reveal significant differences in the number of patients experiencing such effects.

Having analyzed the results of published clinical trials on the application of MSCs in various pathologies, we conclude that this was the highest total dose of intravenous MSCs (1.6 × 10^9^) applied in a clinical study. Although Wilson et al used a single dose of 10 million cells per kilogram in acute respiratory distress syndrome (ARDS), it was a single infusion.^24^ Previously, the highest MSCs dose applied in humans was recorded in the clinical study by Patila et al in patients with ischemic heart failure, in which up to 1.35 × 10^9^ autologous stem cells were administered transmyocardially without any serious adverse events (SAEs).^25^ The issue of safety and tolerance of such high doses is important to consider; although almost all the clinical studies in the field of intravenous MSCs transplantation confirmed the absence of notable adverse effects, most studies used allogeneic cells from one donor, whereas we applied cells from different donors to one and the same patient.^26^ It is not improbable that the higher observed incidence of effects like fever and chills, which are rarely seen in other studies, is related to this fact. Nevertheless, Tzouvelekis et al registered a temperature elevation in 50% of patients after endobronchial administration of 0.5 × 10^6^ per kilogram of body weight adipose‐derived MSCs.^15^ A transitory fever was also one of the most frequent SAEs after intravenous infusions of allogeneic MSCs in the treatment of some nonpulmonary diseases.^27^, ^28^ The other observed adverse effects were similar in incidence between the placebo group and the cell therapy group. One female patient, 73 years of age, suffered an ischemic stroke at day 29 after the first two MSCs infusions; however, we cannot relate this event to the therapy.

The level of mortality did not differ between the study groups: two patients died in each group, and all the cases were the most severe ones with the lowest functional characteristics, were oxygen‐dependent, and had comorbidities including pulmonary hypertension, ischemic heart disease, and chronic obstructive pulmonary disease. Of five included patients with an initial DLCO <25% of predicted and 6MWDT <150 m, four died after four cell (8 × 10^8^) or placebo infusions. We believe that cell therapy might be ineffective at this advanced stage of the disease.

Our data differ from the results of Chambers et al,^14^ who observed a significant increase in 6MWD and a marginal improvement in DLCO and fibrosis score 3 months after a single dose of 1 × 10^6^ per kilogram or 2 × 10^6^ per kilogram placenta‐derived MSCs. In our case, both patients who received 400 × 10^6^ MSCs as well as the patients from the placebo group demonstrated a further decline of pulmonary function during the first 13 weeks of treatment. This may be explained by a more severe disease course in our study than that mentioned in the study of Chambers. Our results from the first 3 months of treatment are closer to the data of Glassberg et al,^16^ who showed a decrease of mean FVC by 1.5% (−4.9% by our data) and DLCO by 7% (−5.2% by our data). In our study, the group receiving the MSCs treatment in general showed a stabilization of DLCO and FVC up to 26 weeks of treatment as well as an increase in the 6MWDT from week 13 and an increase in FVC by week 39 as compared with the placebo group.

We suppose that the effects of MSCs administration are dose‐dependent; indeed, application of different doses (1 million per kilogram, 5 million per kilogram, and 10 million per kilogram) in patients with ARDS resulted in a corresponding reduction of the Lung Injury Score of 30%, 36%, and 45% and of the Sequential Organ Failure Assessment Score of 4%, 23%, and 48%, respectively.^24^ In patients with ischemic heart disease, a dose of fewer than 50 million cells did not significantly affect the estimated parameters, whereas doses of 50 to 100 million and 100 to 250 million cells resulted in a boost of the left ventricular (LV) ejection fraction and LV end‐systolic volume.^29^ This is explainable, because the paracrine effect of transplanted MSCs is currently suggested as their most probable mechanism of action: MSCs and their condition media can cause an antifibrotic effect via suppression of apoptosis of epithelial and endothelial cells, deceleration of epithelial–mesenchymal transition, and suppression of activated fibroblasts and inflammatory response due to their own secretion of anti‐inflammatory cytokines and chemokines, adhesion molecules, growth factors, and so forth.^30^, ^31^, ^32^, ^33^

Interestingly, in the therapy group, no episode of acute exacerbation was registered in any of the patients for the year‐long follow‐up period (Figure 3D). This fact allows an assumption that the MSC therapy with the above‐mentioned paracrine factors may provide the prevention of acute exacerbations. However, this assumption requires further research.

Thus, MSCs may be considered a multicomponent drug, a source of soluble anti‐inflammatory and antifibrotic factors. Correspondingly, an increase in the dose and frequency of cell administration must theoretically boost their efficiency, while simultaneously raising the risk of negative effects. The MSCs administration regimen we studied demonstrated an absence of notable adverse reactions along with a significant suppression of pulmonary function decline, as compared with the previous studies with lower MSCs doses.

Certain concerns with the MSC therapy are related to its potential ability to intensify fibrogenesis in the lungs. Indeed, a possibility has been shown for differentiation of fibrocytes (of the MSC subpopulation circulating in the blood) into fibroblasts/myofibroblasts.^34^ The increase of the MSC level in the bronchoalveolar lavage (BAL) liquid is a predictor of bronchiolitis obliterans development in the transplanted lungs.^35^ In our study, we did not measure the levels of fibrocytes in the blood and MSC in the BAL liquid; however, we did not find radiological signs of fibrosis in the active therapy group.

The HRCT‐based fibrosis score did not show statistically significant differences between the study group and the placebo group. This might be due to either the small number of study participants or the fact that the cell therapy effects are realized predominantly at the functional level. The increase of the lung diffusion capacity as well as the significant increase of 6MWDT may also reflect the latter consideration. One of the mechanisms of such functional improvement may by a systemic angiogenic effect of MSCs and the enhancement of microcirculation both in the remainder of the normal lung tissue and in the skeletal muscle. However, such vascular effects of cell therapy are difficult to estimate using HRCT, which probably explains the functional improvement of patients without notable dynamics of the visualized lung pattern.

The proangiogenic mechanism of the MSC effect is verified by the data obtained in the animal models of diabetes, physical overexertion, and diaphragm dysfunction, demonstrating that administration of different MSC types resulted in augmented angiogenesis in the muscle tissue, increase in the blood flow, and enhancement of the mitochondrial reserve and muscle function.^36^, ^37^, ^38^, ^39^ The proangiogenic potential is also confirmed by the recent proteomic studies showing overexpression of the proteins associated with angiogenesis in the MSCs' secretome, the angiogenic effect being proved with the tube formation assay.^40^ It is of note that many proangiogenic factors are simultaneously anti‐inflammatory; therefore, the classic point of view on the MSCs as inducers of only anti‐inflammatory cytokines probably requires reconsideration.^40^ It should be noted that the proangiogenic MSC effect in patients with IPF is only our hypothesis made in an attempt to explain the obtained positive effects in this pilot study and is based only on the previously published studies of MSCs.

Interestingly, a MSCs mixture from different donors demonstrates a higher immunosuppressive activity than a cell pool from one donor.^41^ This is probably related to a significant individual variability of both the proliferative and immunotropic potentials of MSCs obtained from different donors.^42^ However, because we used in our study young, healthy relatives as donors, we decided to not use the mixture of MSCs from different donors. For those patients to whom we administrated MSCs from nonrelatives, donors for each injection have been randomized. To minimize the risk of using MSCs with “weak” regenerative properties, we conducted the immunophenotype and differentiation analyses of cells from each donor. We also did not use MSCs with low proliferative capacity and MSCs from more than the fifth passage.

All patients in the current study had a higher rate of FVC decline (−10.5%) per year at the moment of inclusion into the study than those in the average population of patients with IPF (−6.2%).^4^, ^10^ Thus, for the first time, we performed a study of the effects of MSCs transplantation in patients with IPF with the most aggressive course of the disease and the poorest prognosis. Moreover, our patients in general belonged to a more severely impaired functional category than those who participated in studies of the antifibrotic drugs nintedanib and pirfenidone, which were approved for IPF treatment.^10^, ^11^ One unusual case, a 33‐year‐old man, a surgeon, with HRCT findings of definite UIP, without a history of smoking and exposure to environmental pollutants, and with negative tests for autoimmunity, underwent a video‐assisted thoracoscopic surgery. The lung biopsy revealed the morphologic pattern of UIP, and MDD confirmed the diagnosis of IPF despite the young age.

The limitations of the current study include a small number of study participants as well as performance of treatment at a single center, which may explain the observed oscillations in the dynamics of certain indices (eg, substitution of an increase in DLCO by a decrease between 26 and 39 weeks) as well as the absence of significant differences in the fibrosis score assessment based on the HRCT data. Furthermore, the procedure was not blind for the researchers. We also could not investigate the distribution and survival of the transplanted cells in the recipients' bodies, their influence on the cytological analysis of the BAL fluid, or biomarkers of the fibrotic activity.

## 5 CONCLUSION

We have shown in this study that high cumulative dose of transplanted bone marrow–derived MSCs is safe and well tolerated. The safety of the MSCs therapy has been proved by the fact that even application of an extremely high dose of stem cells (the highest one on the record) has not been associated with any significant adverse effects, except for a transitory fever during the first day after the infusion noted in 4 out of 10 patients. During the treatment period, patients in the main group had their lung function increased, as compared with the baseline and the same parameters in the placebo group, in which the continued decline of lung function was observed. Thus, we state the safety, tolerability, and potential benefits of greater doses of MSCs than those used earlier in patients with IPF and other pulmonary diseases, and these findings can move future trials toward a new step in stem cells transplantation.

## CONFLICT OF INTEREST

V.K. and V.B. declared researched funding from Federal Medical and Biological Agency, Order 20.001.13.800. The other authors indicated no potential conflict of interest.

## AUTHOR CONTRIBUTIONS

A.A.: conception and design, manuscript writing, reviewed the manuscript; I.K., O.D., A.S.: patient recruiting and observation, collection and assembly of data, reviewed the manuscript; V.L.: collection and assembly of data, HRCT performing, reviewed the manuscript; V.R., V.K.: provision of MSCs for the transplantation, reviewed the manuscript; M.K., A.N.: MSCs characterization, assembly of data, reviewed the manuscript; V.B.: MSCs quality control, administrative support, manuscript writing, reviewed the manuscript.

## Supporting information


**Appendix S1**: Supporting information

## Data Availability

The data that support the findings of this study are available on request from the corresponding author.
