# Supplementary material for: First‐in‐human high‐cumulative‐dose stem cell therapy in idiopathic pulmonary fibrosis with rapid lung function decline
Source: Stem Cells Transl Med. 2019 Oct 15;9(1):6–16. doi: 10.1002/sctm.19-0037 (PMC6954714; doi:10.1002/sctm.19-0037)

Supplementary materials

FIRST-IN-HUMAN HIGH CUMULATIVE DOSE STEM CELL THERAPY IN IDIOPATHIC PULMONARY FIBROSIS WITH RAPID LUNG FUNCTION DECLINE

Running head: STEM CELL THERAPY IN IDIOPATHIC PULMONARY FIBROSIS

Alexander Averyanov[1,2], Irina Koroleva[1], Mikhail Konoplyannikov[1], Veronika Revkova[1], Victor Lesnyak[1], Vladimir Kalsin[1], Olesya Danilevskaya[1,2], Alexey Nikitin[1,2], Anna Sotnikova[1,2] and Vladimir Baklaushev[1,2*]

[1]Federal Research and Clinical Center of Federal Medical-Biologic Agency, 28 Orekhovy Blvd., 115682 Moscow, Russia;

[2]Pulmonology Scientific Research Institute under Federal Medical- Biologic Agency FMBA of Russia

Inclusion Criteria

Subjects must satisfy the following criteria to be enrolled in the study:

- Male and female patients 20-80 years old
- Diagnosis of idiopathic pulmonary fibrosis based on:
- Clinical symptoms > 12 months duration,
- Histologically diagnosed or diagnostic chest HRCT features of usual interstitial pneumonia as outlined below
- Forced Vital Capacity (FVC) ≥ 50% of predicted and Diffusing Lung Capacity (DLCO) ≥30% of predicted
- Loss more than 10% of baseline of FVC or DLCO during the last 12 months
- Signed informed consent.

Exclusion Criteria

The presence of any of the following will exclude a subject from enrollment:

- Diagnosis of an interstitial lung disease other than idiopathic pulmonary fibrosis (sarcoidosis, pulmonary alveolar proteinosis, lymphangioleiomyomatosis, pulmonary amyloidosis, exposure-related lung disease etc)
- Obstructive lung disease: FEV1/FVC < 0.70
- Clinically significant medical condition, in the opinion of the investigator, may compromise the results of the study
- Evidence of active infection within 4 week prior to enrollment
- History of malignancy < 5 years prior to enrollment
- Unable to cooperate with any study procedures
- Pregnant or breast-feeding
- Treatment with antiinflammatory or antifibrotic drugs including high doses oral steroids (more than 30 mg/daily), cytostatic drugs, pirfenidone, nintedanib, D penicillamine, colchicine, tumor necrosis factor α blockers, imatinib, interferon γ, monoclonal antibodies < 6 months prior to randomization.
- Active listing for transplant of any organ.
- Known history of alcohol abuse within 1 year prior to enrollment

- Participation in another clinical trial

Suppl. Table 1. Summary of Criteria for Diagnosis of Idiopathic Pulmonary Fibrosis Based on High Resolution Computed Tomography and Surgical Lung Biopsy

	Surgical Lung Biopsy Not Available	Pathology: Definite UIP	Pathology: Probable UIP	Pathology: Possible UIP	Pathology: Inconsistent with UIP or Not Classifiable
HRCT by central review: UIP Pattern	Eligible	Eligible	Eligible	Eligible	NOT Eligible
HRCT by central review: Possible UIP Pattern	NOT Eligible	Eligible	Eligible	NOT Eligible	NOT Eligible
HRCT by central review: Inconsistent with UIP Pattern	NOT Eligible	NOT Eligible	NOT Eligible	NOT Eligible	NOT Eligible

Suppl. Figure 1: Power of the analysis for FVC

Independent Sample t-Test: Power Calculation Two Means. t-Test. Ind. Samples (H0: Mu1 = Mu2) Power vs. N (Es = 1.25496. Alpha = 0.05. N1 = N2 = N)

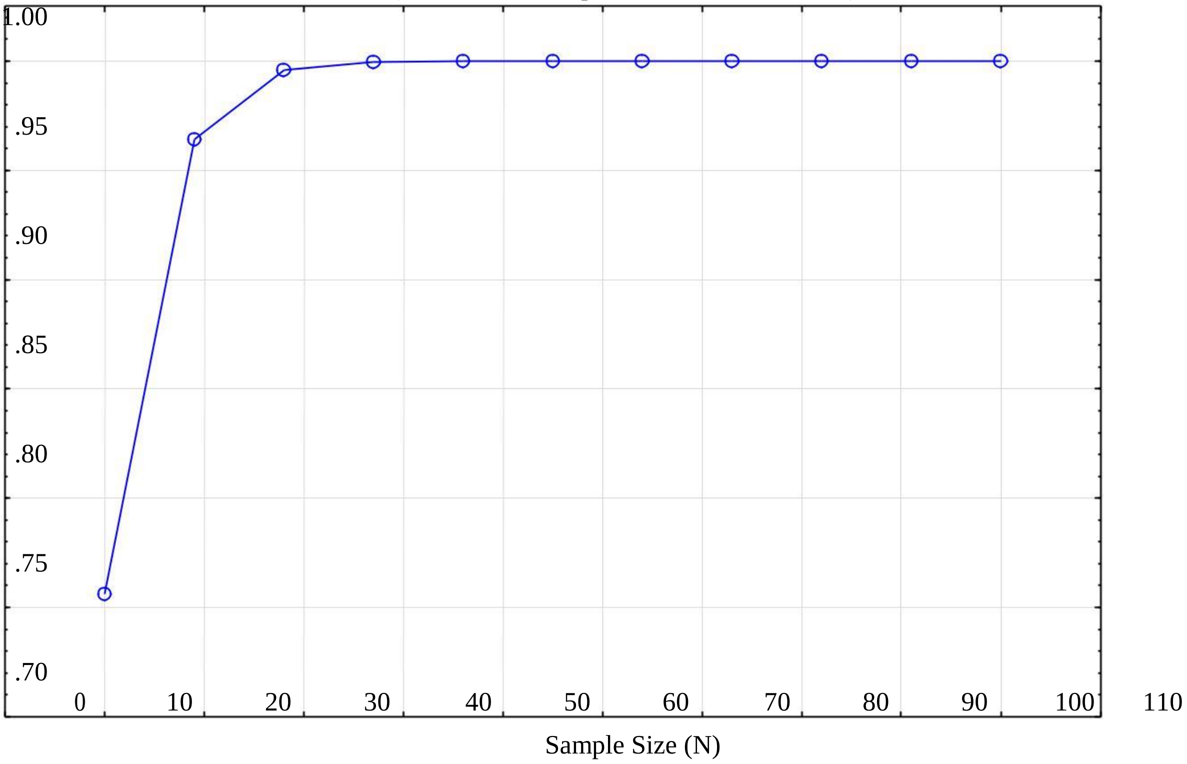

Suppl. Figure 2: Power of the analysis for DLCO

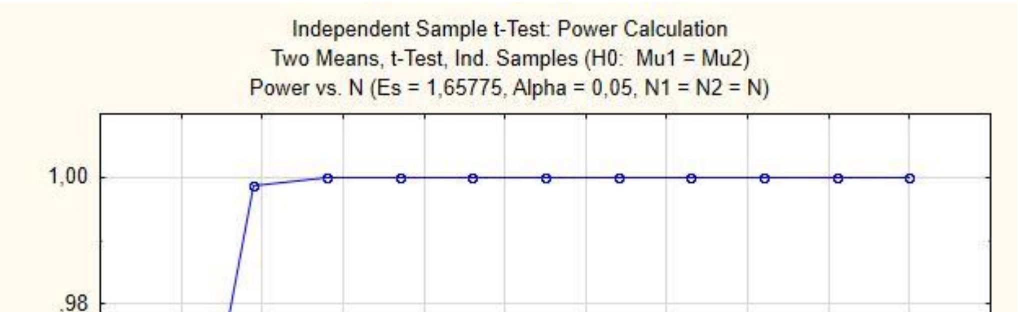

Supplement: Supplementary file 1 — Appendix S1: Supporting information [file SCT3-9-6-s001.pdf]
